# Nano-Pulse Stimulation Ablates Orthotopic Rat Hepatocellular Carcinoma and Induces Innate and Adaptive Memory Immune Mechanisms that Prevent Recurrence

**DOI:** 10.3390/cancers10030069

**Published:** 2018-03-13

**Authors:** Brittany P. Lassiter, Siqi Guo, Stephen J. Beebe

**Affiliations:** Frank Reidy Research Center for Bioelectrics, Old Dominion University, 4211 Monarch Way, Suite 300, Norfolk, VA 23508, USA; blassite@odu.edu (B.P.L.); s2guo@odu.edu (S.G.)

**Keywords:** effector memory T-cells, central memory T-cells, memory precursor effector T-cells, short-lived effector T-cells, NK cell receptors, NKT cells, T-regulatory cells

## Abstract

Nano-pulse stimulation (NPS), previously called nsPEFs, induced a vaccine-like effect after ablation of orthotopic N1-S1 hepatocellular carcinoma (HCC), protecting rats from subsequent challenges with N1-S1 cells. To determine immunity, immune cell phenotypes were analyzed in naïve, treated and protected rats. NPS provides a positive, post-ablation immuno-therapeutic outcome by alleviating immunosuppressive T regulatory cells (Treg) in the tumor microenvironment (TME), allowing dendritic cell influx and inducing dynamic changes in natural killer cells (NKs), NKT-cells and T-lymphocytes in blood, spleen and liver. NPS induced specific increases in NKs and NKT-cells expressing CD8 and activation receptors CD314-NKG2D and CD161 (NK1.1) in the TME after treatment, as well as some variable changes in CD4+ and CD8+ effector (Tem) and central memory (Tem) lymphocytes in blood and spleen. After orthotopic challenge, CD8+ T-cells were cytotoxic, inducing apoptosis in N1-S1 cells; additionally, in contrast to post-treatment immune responses, CD4+ and CD8+ memory precursor effector cells (MPECs) and short-lived effector cells (SLECs) were present, while still including CD8+ CD161 NK cells, but not involving CD8+ CD314-NKG2D+ NKs. This immunity was N1-S1-specific and was sustained for at least 8 months. NPS vaccinates rats in vivo against HCC by activating innate and adaptive immune memory mechanisms that prevent HCC recurrence.

## 1. Introduction

The liver is the largest internal organ in the body and serves a wide range of functions related to digestion, nutrient storage, metabolism, detoxification and immunity among others. The liver has a dual blood supply from the hepatic artery providing fresh oxygenated blood and from the hepatic portal vein from the digestive tract delivering intestinal nutrients, bacterial products, environmental toxins and drugs. The blood supply courses through a specialized sinusoidal structural design that maximizes interactions between immune cells and pathogens. Chronic hepatocyte injury and efferocytosis of dead and dying cells leads to production of pro-inflammatory cytokines, linking hepatocyte apoptosis and liver fibrosis [1,2,3,4]. In contrast to other homeostatic functions where programmed cell death is a silent, non-inflammatory process [5], programmed cell death and inflammation, as part of liver homeostasis, is unique to hepatocytes. Chronic liver inflammation promotes genetic instability that transforms cells, develops resistances to apoptosis and immune surveillance, and facilitates hepatocellular carcinoma (HCC) resistances to chemotherapeutic agents [5,6]. Symptoms and diagnoses of HCC generally occur at advanced stages, when prognoses are usually poor, so HCC incidence and mortality rates are nearly equal.

Liver cancer is the fifth-most common cancer, with over half a million cases every year [7], and about 75% of these are HCC [8]. A recent report estimated that instances of liver cancer will continue to increase through years 2020 and 2030 in the United States, becoming the number three cause of cancer deaths by 2030, behind lung and pancreatic cancer [9]. HCC often begins with cirrhosis, progressing to adenoma and dysplastic nodule formation [10]. Continual proliferation and tissue regeneration leads to uncorrected mutations, giving rise to altered stem cells with greater chances for further changes causing tumor progression [11].

Although not generally considered part of the immune system, like the spleen, lymph nodes and thymus, the liver is in fact an immunological organ [12]. The liver’s innate lymphoid immune system forms an immunological barricade to pathogen intrusion by distinct sets of antigen-presenting cells (APC), including liver sinusoidal endothelial cells (LSEC), Kupffer cells, stellate cells and dendritic cells (DCs). Also included in this defense system are enriched levels of natural killer (NK) cells and NKT-cells, as well as T-lymphocytes with enhanced levels of CD8+ cells [13]. Given the liver’s position in immune surveillance and innate immunity as first responders to pathogens and toxic insults [12,14,15], tight regulation between tolerance and immunity must be maintained for differential responses to non-pathogenic and pathogenic molecules [16,17].

For more than a decade, applications of nanosecond-pulsed electric fields (nsPEFs), now called Nano-Pulse Stimulation (NPS), have shown ablation efficacy against a wide range of tumor types, including HCC [18]. While not yet clinically available, NPS provides an additional HCC therapy to other technologies using electric fields, which include electrochemotherapy (ECT) [19] and irreversible electroporation [20], both of which have shown benefit for HCC in humans. Results presented here indicate that NPS may provide additional significant advantages beyond ablation for HCC treatment. NPS induced a post-HCC ablation, vaccine-like effect in rats [21] and triggered CD8+-dependent inhibition of secondary HCC tumors in rats [22]. More recently, successful NPS treatment of mammary cancer induced a vaccine-like effect that prevented breast cancer metastasis to spleen, lung and liver and induced robust immune responses in orthotopic mammary cancer in mice [23]. NPS has also been shown to induce release of immunogenic danger-associated molecular patterns (DAMPs) in several tumor cell types [22,23,24]. The suspected immune response behind the vaccine-like effect protecting rats from N1-S1 HCC challenge after ablation by NPS has not been investigated until now.

In this report, the hypothesis was tested that our previous finding of a protective, vaccine-like effect after NPS ablation of rat HCC [21] was induced by immune responses observable in blood, spleen and liver. We show that this post-ablation, vaccine-like effect is specific to N1-S1 HCC, not rat H4IIE HCC, and is in fact mediated by robust innate and adaptive immune responses. Different adaptive and innate immune responses are recalled in post-NPS ablation after orthotopic challenge of N1-S1 cells. After NPS, rapid increases of specific NK and NKT-cell subsets appear in liver, as well as by CD8+ and CD4+ effector memory and central memory T-cell responses in the HCC tumor microenvironment (TME), spleen and blood. The immune responses to post-ablation challenge with N1-S1 cells includes cytotoxic CD8+ T-cells and an immune response distinctly different from the post-treatment response. These memory responses last at least 8 months, and an abscopal effect can prevent secondary tumor growth when tumors are of limited size. 

## 2. Results

### 2.1. NPS Induces an Adaptive Immune Response in Blood and Spleen after HCC Ablation

NPS Induces Changes in CD4+ and CD8+ Tem and Tcm Lymphocytes in Blood and Spleen

**Figure 1:**
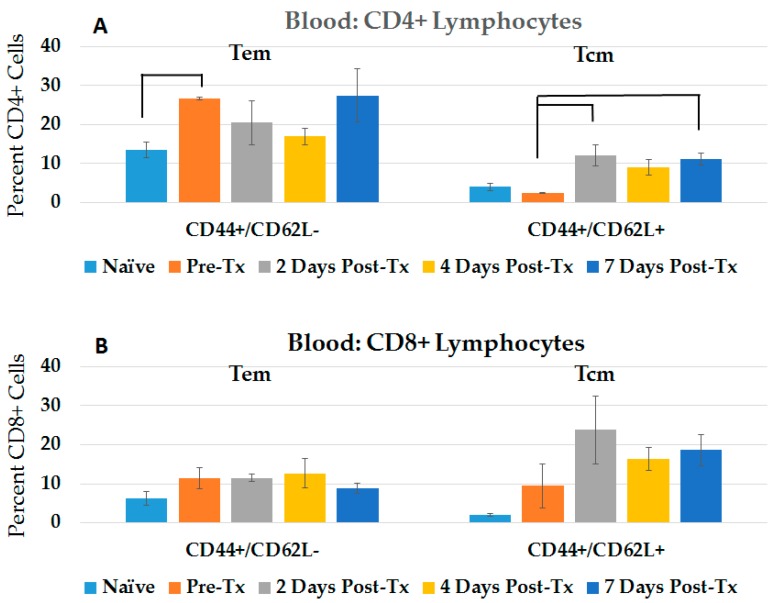
Circulating CD4+ and CD8+ Tem and Tcm increase in response to NPS. Blood was taken from rats at various times post-Tx using Institutional Animal Care and Use Committee (IACUC) approved methods. Lymphocytes were isolated using Ficoll, then stained with CD4, CD8, CD44 and CD62L; analysis was done on CD4+ cells (**A**) and CD8+ cells (**B**). Error bars = standard error of the mean (SEM); Naïve, 2 Days, 4 Days *n* = 4; Pre-Tx *n* = 2; 7 Days *n* = 5. CD44+/CD62L−, CD44+/CD62L+ 2 Days Post-Tx: CD44+/CD62L+ 7 Days Post-Tx: *p* < 0.01.

**Figure 2:**
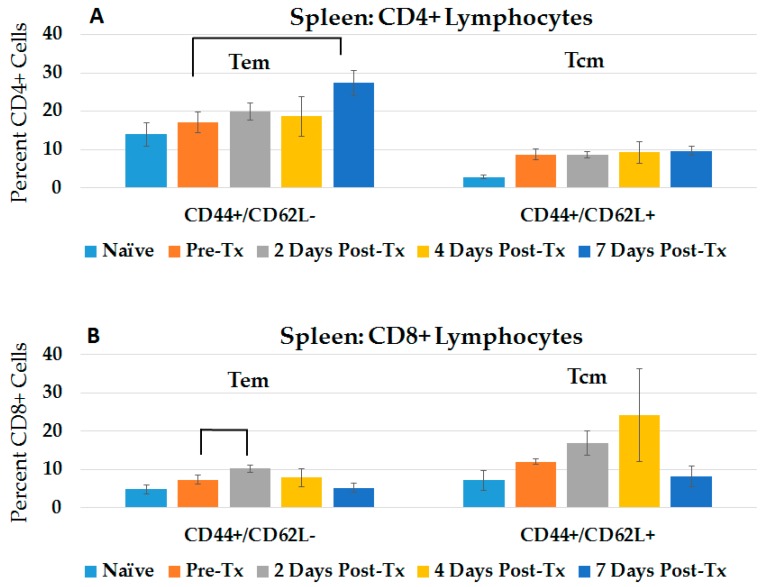
NPS induces increases in splenic CD4+ Tem and CD8+ Tem and Tcm after treatment. Lymphocytes were isolated from spleens at various times post-treatment, then stained with CD4, CD8, CD44 and CD62L; analysis was done on CD4+ cells (**A**) and CD8+ cells (**B**). Error bars = standard error of the mean (SEM); Naïve, 2 Days, 4 Days *n* = 4; Pre-Tx n=2; 7 Days *n* = 5. A. CD44+/CD62L− 7 Days Post-Tx: *p* = 0.02. B. CD44+/CD62L+ 2 Days Post-Tx: *p* < 0.004; connecting lines *p* < 0.05.

Central memory (Tcm) and effector memory (Tem) cells are distinguished from naïve T-cells by the presence of CD44, which is involved in lymphocyte activation. Tcm and Tem cells are differentiated by the presence and absence, respectively, of CD62L (L-selectin), which functions as a lymph node adhesion/homing receptor. To determine if NPS induced an immune response after treatment of rat orthotopic N1-S1 HCC tumors, CD4+ and CD8+ Tem cells (CD44+ CD62L−) and Tcm cells (CD44+ CD62L+) were analyzed in blood (Figure 1) and spleen (Figure 2). In pre-treated tumor-bearing rats, only CD4 Tem cells were elevated above naïve controls. During the 7-day post-treatment study, only CD4+ Tcm were significantly elevated (5.2-fold, day 2), although CD8+ Tcm tended to be higher (1.7–2.5-fold), but not with statistical significance. After NPS treatment, CD4+ and CD8+ Tem cells were no different from pre-treated tumor-bearing rats. Over the 7-day study in spleen, CD4+ and CD8+ Tem cells were significantly elevated (1.6-fold day 7; 1.4-fold, day 2, respectively), while CD8+ Tcm showed a time-dependent increase to about 2-fold, but not with statistical significance. Generally, within the first week after NPS treatment, blood and spleen levels of adaptive T-cells showed trends towards increases, but variability among individual animals did not always allow statistical significance.

CD4+ and CD8+ memory precursor effector cells (MPECs: CD127+ KLRG1−) and short-lived effector cells (SLECs: CD127− KLRG1+) were absent or present at very low unchanging levels in response to NPS treatment during the study in spleen and blood.

### 2.2. NPS Induces Innate and Adaptive Immune Responses in Liver after HCC Ablation

#### 2.2.1. Resolution of Treg Immunosuppression and DC Infiltration in the TME after NPS

In rat liver studies, analyses included the tumor-bearing middle liver lobe as the TME. In two individual studies, all liver lobes expressed similar cell levels and phenotypes, indicating that the liver functioned in unison with the presence of treated and untreated N1-S1 HCC tumors.

**Figure 3:**
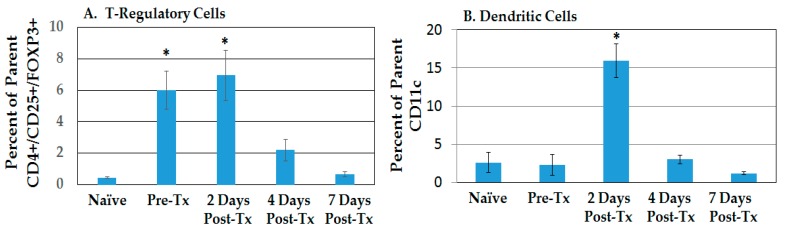
Immunosuppressive Tregs dissipate while dendritic cells infiltrate the TME in response to NPS. Lymphocytes were isolated from the liver of naïve rats and from rats at various times post-Tx and stained with either (**A**) FOXP3/CD25/CD4 (naïve *n* = 4, others *n* = 2) or with (**B**) CD11c (Naïve *n* = 5, Pre-Tx *n* = 4, 2 days n=4, all others *n* = 6); error bars = SEM. * *p* < 0.05.

We analyzed hepatic immunosuppressive T-regulatory cells (Tregs) with CD4+ CD25+ FoxP3 phenotypes (Figure 3A) and dendritic cells (DCs) with CD11c phenotype (Figure 3B). Compared to naïve rats, Tregs significantly increased over 13–15-fold, before regressing to naïve levels by day 7 post treatment. By the 2nd day post treatment, DCs were significantly greater (7.0-fold), compared to pre-treated rats before the Treg level had decreased. The alleviation of the immunosuppressive TME and the entry of DCs show indications that immune responses can be initiated.

#### 2.2.2. HCC and NPS Induce Multiple Phenotypic Changes in Liver Tumor-Infiltrating Lymphocytes (TILs)

**Figure 4:**
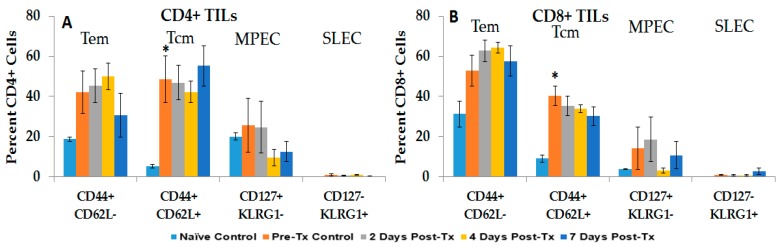
Tumor-infiltrating lymphocytes (TILs) change phenotypes in response to NPS in the liver. Lymphocytes were isolated from rat livers (tumor bearing lobe) at various times post-Tx. Cells were stained with CD4, CD8, CD44, CD62L, CD127 and KLRG1. Analyses were done on CD4+ cells (**A**) and CD8+ cells (**B**). Error bars = standard error of the mean (SEM); Naïve *n* = 4, Pre-Tx *n* = 5; others *n* = 6; * *p* < 0.05.

Figure 4 shows CD4+ (A) and CD8+ (B) tumor-infiltrating lymphocytes (TILs) in the liver TME. Generally, CD4+ and CD8+ TIL phenotypes were elevated above naïve levels in pre-treated tumor-bearing rats, except that SLECs were essentially absent; CD4+ and CD8+ Tcm cells were significantly increased. After NPS treatment, no TIL phenotype was elevated above the level for pre-treated tumor-bearing rats; however, levels varied due to large variations among different animals. While MPECs were absent in blood and spleen (text with Figure 1 and Figure 2), they were present in the liver TME, but with variable levels during the week following NPS treatment. Nevertheless, the presence of CD127, the IL7Rα chain, indicates the presence of cells with proliferative and anti-apoptotic potential for memory T-cells [25]. Given that these TILs are in the presence of Treg-dominated immunosuppressive TME until day 4 (Figure 3), they are likely anergic or ineffective, as indicated by the failure of tumor-bearing rats to survive without NPS treatment. It is likely that after NPS, these cells and others like them are recruited to the treatment zone and become cytotoxic against tumor cells and participate in immune responses.

#### 2.2.3. Dynamic Changes in Liver NK Cells, NKT-Cells and T-Lymphocytes in the TME after NPS

**Figure 5:**
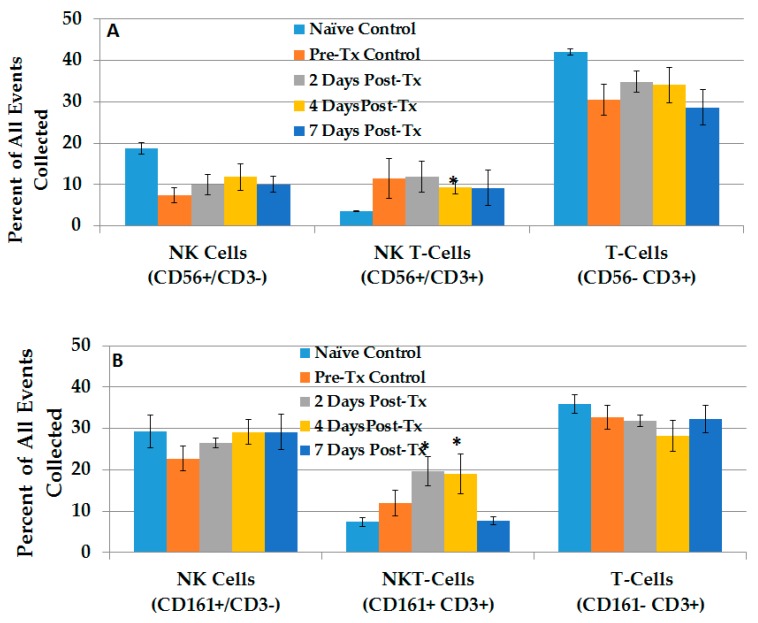
Differential analysis of natural killer cells with CD56 or CD161 as the defining marker. Lymphocytes were isolated from rat livers (tumor bearing lobe) at various times post-Tx. Cells were stained with CD56, CD161, CD3, CD8a and CD314-NKG2D. Analyses were done on CD56+/CD3− cells (**A**) CD161+/CD3− (**B**). Error bars = standard error of the mean (SEM); Naïve *n* = 4, Pre-Tx *n* = 5; others *n* = 6. * *p* < 0.05.

To analyze NK cells, NKT-cells and T-cells in liver, we used CD3 and CD56 phenotypes in one approach and CD3 and CD161 phenotypes in another approach. This latter approach is more commonly used in mice, but was used here for completeness, because these phenotypes have not been investigated in rats. NK cells are CD3− and CD56+ or CD161+; NKT-cells are CD3+ CD56+ or CD161+ and T-cells are CD3+ CD56− or CD161−. Supplementary Figure S1 shows a typical flow cytometric analyses of liver using the CD3 and CD56 phenotype approach. Natural killer (NK) cells defined as CD56+ CD3− (upper left quadrant), NKT-cells defined as CD56+ CD3+ (upper right quadrant), and T-cells defined as CD56− CD3+ (lower right quadrant) were analyzed in naïve pre-treated and rat livers 2, 4, and 7 days after NPS treatment. These cells were also analyzed using the CD3 and CD161 phenotype approach in the same way. Several of these experiments provided the bases for analyses of NK, NKT- and T-cells using the CD3 CD56 approach, as shown in Figure 5A, and the CD3 CD161 approach, as shown in Figure 5B.

In Figure 5A, using the CD56 and CD3 approach, rat liver NK cell levels were relatively high in naïve rat livers, while NK levels tended to be decreased in pre-treated rats and remain relatively constant following NPS treatment, with no significant differences among them. NKT-cell levels were significantly elevated 2.6-fold (day 4) above naïve levels, and remained elevated during the 7-day study; however, treated levels were not elevated compared to pre-treated levels. Elevated levels of T-cells were present in rat livers under all conditions.

When these cells were analyzed using the CD161 and CD3 approach (Figure 5B), NK cell levels tended to be higher than the CD56 CD3+ phenotype, but their levels did not change before or after treatment. The CD161− CD3+ T-cell phenotype was also elevated before and after NPS treatment. Pre-treated CD161+ CD3+ NKT-cells were elevated slightly, and then on day 4 were elevated significantly above naïve levels, but were not elevated above pre-treated levels. CD161 is known to be expressed predominantly on T-cells with memory phenotypes [26], so its presence is likely important. Yet, a simple analysis of CD3, CD56 and/or CD161 phenotypes did not show changes in the first week after NPS treatment. Nevertheless, a more in-depth phenotypic analysis did show significant differences.

#### 2.2.4. NPS Activates Distinct NK and NKT-Cell Phenotypes in the TME

**Figure 6:**
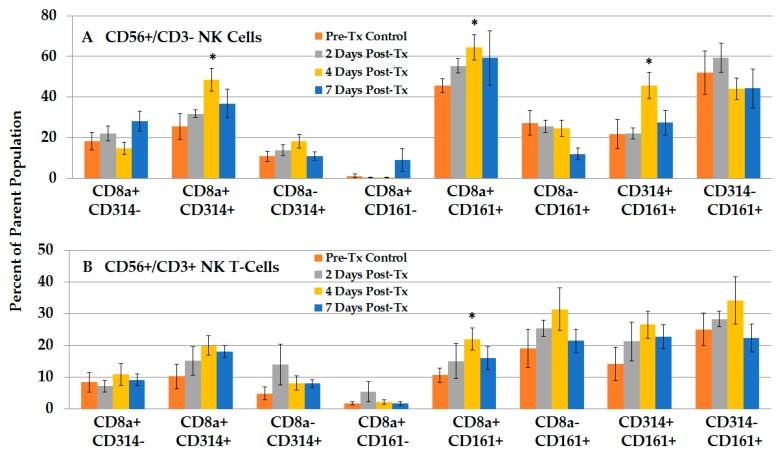
NPS induces an increase in various phenotypic subsets of NK and NKT-cells. Liver lymphocytes were analyzed with CD8a, CD314 (NKG2D) and CD161 on (**A**) NK cells (CD56+/CD3−) and (**B**) NKT-Cells (CD56+/CD3+). Naïve *n* = 4, Pre-Tx *n* = 5; others *n* = 6; error bars = SEM. * *p* < 0.05.

Immune cells are generally more abundant and perhaps more specialized in the liver. To further investigate NK, NKT- and T-cells in liver, we focused on the CD3 CD56 phenotype approach because it is more commonly used in rat immune studies. Figure S2 shows an additional strategy using CD3− CD56+ NK cells for expression of CD8, CD314-NKG2B and CD161 (NK1.1) in naïve rats (left panels) livers and treated, tumor-regressing rat livers (right panels). CD314-NKG2D is a well-characterized activation receptor for detection and elimination of transformed cells [27], and CD161 is another known activation marker reported to be present on memory T-cells [26]. Naïve rat NK phenotypes included expression of CD11b (bottom left panel) and relative low levels of CD8 and CD161 (18.4%), as well as low levels of CD8 and CD314-NKG2D (17.9%). After NPS, the liver NK cell population underwent several NPS-induced phenotypic changes, with decreased expression of CD11b (bottom right panel) and increased CD8+ NK phenotypes with CD161 (57.5%) and CD314-NKG2D (57.3%) activation receptors. The same strategies were used to analyze CD3+ CD56+ NKT-cells and CD3+ CD56− T-cells (not shown). Figure 6A,B shows these studies for NK and NKT-cells, respectively. Consistent with the results shown in Figure 4, the changes in T-cell CD3+ CD56- phenotype were less remarkable and are shown in Figure S3. 

Figure 6A shows that NPS treatment induced a time-dependent increase in NK cells after treatment, with significant increases in CD8 and CD314-NKG2D (1.9-fold, day 4), as well as NKs that expressed CD8 CD161 (1.4-fold, day 4). NK cells with CD314-NKG2D and CD161 were also significantly increased by day 4 post NPS treatment. Figure 6B shows that NKT-cells also showed NPS-induced increases in CD8+ CD314-NKG2D+ phenotype (2-fold, day 4), albeit not significantly, and significant increases in CD8+ CD161+ phenotype (1.4-fold, day 4). In general, expression of these markers was higher in NKs than in NKT-cells. Unlike NK cells, NKT-cells also showed a trend toward increased levels of subsets without CD8, with or without CD314-NKG2D and CD161. Thus, in order to see dynamic changes of NPS-induced effects on NK and NKT-cells, analyses of subsets with CD8, CD314-NKG2D and CD161 phenotypes were needed. 

Our analysis of Tem and Tcm T-cells in Figure 5 did not include an analysis of CD3+ CD56− T-cell phenotypes considering CD314 or CD161 expression. Figure S3 shows that most T-cells defined as CD3+ CD56− were more CD8a+. Those 8a T-cell subsets tended to decrease with time after treatment, regardless of their expression of CD314-NKG2D or CD161. One T-cell subset that was CD8a− CD161+ increased 2 days (2.0-fold) and 4 days (2.7-fold) after NPS, suggesting an activated phenotype. These results suggest that liver CD8+ T-cells that express CD314-NKG2D or CD161 are relatively rare and are likely less important for NPS-induced tumor elimination. 

### 2.3. Immune Responses Mediating the Protective, Vaccine Effect after Challenge Injections

#### 2.3.1. CD4+ and CD8+ Immune Response in Blood in the Post-Challenge, Vaccine Effect

**Figure 7:**
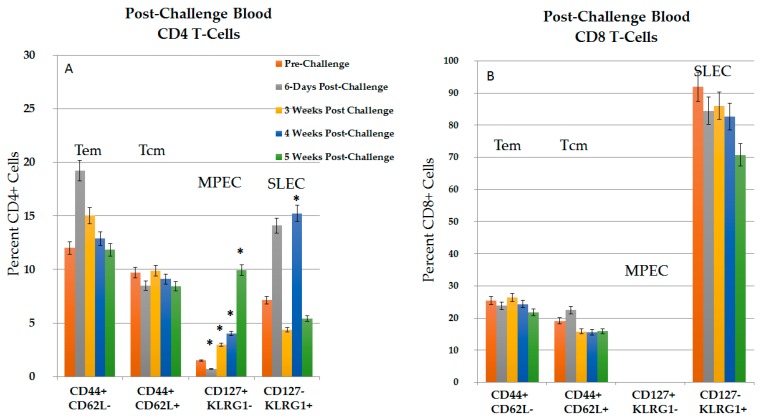
Circulating T-Cells post-challenge show dynamic changes in CD4+ cells. Blood was harvested at various times post-challenge. Lymphocytes were isolated using Ficoll and then stained with CD4, CD8, CD44, CD62L, CD127 and KLRG1. Analyses were done on CD4+ cells (**A**) and CD8+ cells (**B**). *n* = 9, error bars = SEM. * *p* < 0.05.

To analyze the immune response during vaccine-like effect, blood was analyzed for various lymphocyte phenotypes at various times before and after challenge (Figure 7). None of these rats had developed tumors at nine weeks after the challenge injections. In general, changes in CD4+ phenotypes were more dynamic than in CD8+ phenotypes. Compared to pre-challenged rats, there was an increase in CD4+ Tem on day 6 (1.6-fold), followed by a time-dependent decrease to pre-challenge levels 4–5 weeks after challenge. CD8+ Tem cell levels increased in pre-challenged rats 4.1-fold above naïve levels (25.5%, Figure 7B vs. 6.3% in naïve rats, see Figure 1B) and remained elevated, but did not change for 5 weeks in the post-challenge study. CD4+ and CD8+ Tcm cells were also elevated above naïve levels (see Figure 1A,B, respectively), but did not show significant changes after challenge. Thus, after NPS-induced tumor clearance, protected rats expressed elevated levels of CD4 and CD8 Tem and Tcm lymphocytes. Except for CD4+ Tem cells, these did not increase after challenge injections.

The subset of MPECs that up-regulate and retain the IL-7Rα, CD127 form functional memory cell pools, while SLECs that express KLRG1 are effector cells, but are terminally differentiated. After NPS treatment, CD4+ and CD8+ MPEC (CD127+ KLRG1−) and SLEC (CD127- KLRG1+) cells were present at very low levels (data not shown from Figure 1 and Figure 2), suggesting that they do not play a role in the post-treatment immune response. In contrast, after challenge, there was a time-dependent 6.6-fold increase above pre-challenge levels in CD4+ MPECs, with significant increase in weeks 3–5 (Figure 7A). However, no CD8+ MPECs were observed post-challenge (Figure 7B)*.* CD4+ SLECs, which are absent after NPS, increased 2.1-fold above pre-challenge levels at 6 days post-challenge, and exhibited significant increases in 4 weeks post-challenge (Figure 7A). Levels of SLECs were consistently low at 3 weeks post-challenge, suggesting that terminally differentiated SLECs fluctuate during response to challenge. Interestingly, CD8+ SLECs were present in elevated levels before and after challenge injections (Figure 7B). It appears that MPEC and SLEC lymphocytes play a greater role in the vaccine-like, recall response to challenge than they do in response to initial tumor initiation.

#### 2.3.2. CD4+ and CD8+ Immune Responses in Spleen and Liver during the Post-Challenge Vaccine-Like Effect

**Figure 8:**
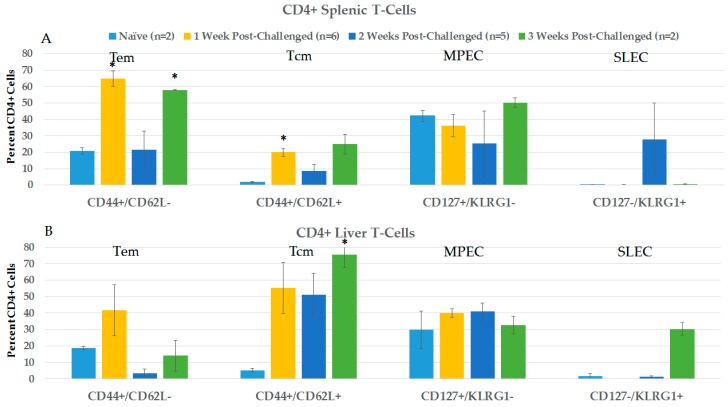
Spleen and liver CD4+ T-Cell analysis post-challenge. Lymphocytes were isolated at various times post-challenge from the spleen (**A**) and liver (**B**), and then stained with CD4, CD8, CD44, CD62L, CD127 and KLRG1. Analyses were done on CD4+ cells. Naïve and 3 weeks *n* = 2, 1 week *n* = 6, and 2 weeks *n* = 5; error bars = SEM. * *p* = 0.05.

Figure 8 shows changes in CD4+ lymphocyte phenotypes in spleen (8A) and liver (8B) in the first 3 weeks after protected rats were orthotopically challenged with N1-S1 cells. In general, CD4+ phenotypes in spleen and liver did not show significant differences from naïve rats after challenge, except in spleen for Tem cells in weeks 1 and 3, and Tcm in week 1, and in liver for Tcm 3 weeks after treatment. Unlike in blood after challenge, CD8+ MPECs are present in the spleen and liver of pre-challenge rats at relatively high levels that remain three weeks after challenge. Additionally, in contrast to blood, SLE cells were essentially absent or at relatively low, variable levels in the spleen and liver after challenge.

**Figure 9:**
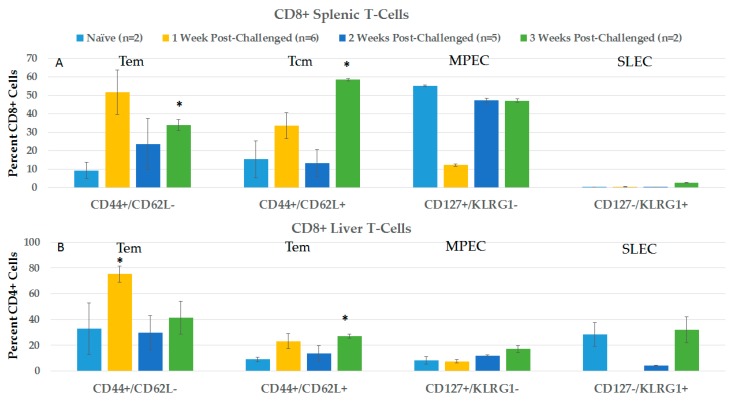
Spleen and liver CD8+ T-Cell analysis post-challenge. Lymphocytes were isolated at various times post-challenge from spleen (**A**) and liver (**B**) and then stained with CD4, CD8, CD44, CD62L, CD127 and KLRG1. Analyses were done on CD8+ cells. Naïve and 3 weeks *n* = 2, 1 week *n* = 6 and 2 weeks *n* = 5; error bars = SEM. * *p* < 0.05.

Figure 9 shows changes in CD8+ lymphocyte phenotypes in spleen (9A) and liver (9B) in the first 3 weeks after challenge. Significant increases were seen in spleen CD8 Tem and Tcm cells 3 weeks after challenge and in liver CD8+ Tem and Tcm cells 1 week and 3 weeks, respectively, after challenge. MPECs were relatively elevated in the spleen and low in the liver before and after treatment. SLECs were very low in the spleen and variable in the liver before and after treatment.

#### 2.3.3. Splenic CD8+ Lymphocytes Are Cytotoxic after NPS-Induced Protection

**Figure 10:**
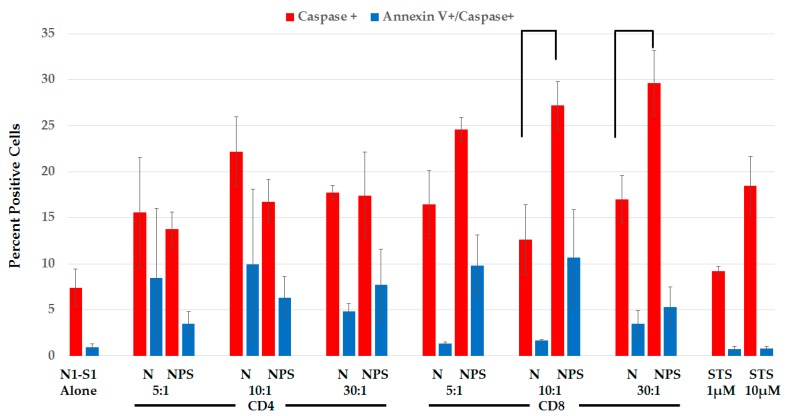
Splenic CD8+ T-cells are more cytotoxic than splenic CD4+ T-cells. T-cells isolated from spleens of rats 2 weeks post-NPS. N1-S1 cells were incubated with separated CD4+/CD8+ T-cells for 16 h and prepared for flow cytometry. All analysis was done on the CellTrace+ N1-S1 cells. *n* = 3 and error bars = SEM. Connecting lines *p* < 0.05.

To determine possible T-lymphocyte cytotoxicity after challenge, we isolated CD4+ lymphocytes (92% pure) and CD8+ lymphocytes (96% pure) from spleens of naïve rats and rats two weeks after treatment with only 2.2 and 1.4% cross contamination, respectively (Figure S4). Isolated lymphocytes were incubated with Cell Tracker Violet-labeled N1-S1 cells, which were analyzed for active caspases and Annexin-V binding as apoptosis markers 24 h later (Figure 10). N1-S1 cell percentage for active caspases (red bars) and caspases plus Annexin-V (blue bars) are indicated on the Y-axis when lymphocytes to N1-S1 ratios were 5:1, 10:1 and 30:1, as shown on the X-axis. CD4+ lymphocytes showed no increases in cytotoxicity; there were no increases in either indicators of NPS N1-S1 cells (NPS) compared to non-NPS N1-S1 cells (N). In contrast, compared to non-treated N1-S1 cells, CD8+ lymphocytes from NPS treated rats exhibited 1.5-, 2.2- and 1.7-fold more active caspase positive N1-S1 cells, respectively, and 7.3-, 6.3- and 1.5-fold more caspase/Annexin-V double positive N1-S1 cells, respectively. However, statistically significant cytotoxicity was only observed in CD8+ T-cells for increases in caspase activity when the ratios between lymphocytes and N1-S1 cells were 10:1 and 30:1. These results indicate that CD8+, but not CD4+, lymphocytes from spleens of protected rats after challenge are cytotoxic towards N1-S1 cells treated with NPS compared to cells not stimulated by NPS.

#### 2.3.4. Distinct Subsets of NK Cells in Liver and Spleen in NPS-Induced Post-Challenge Vaccine-Like Effect

**Figure 11:**
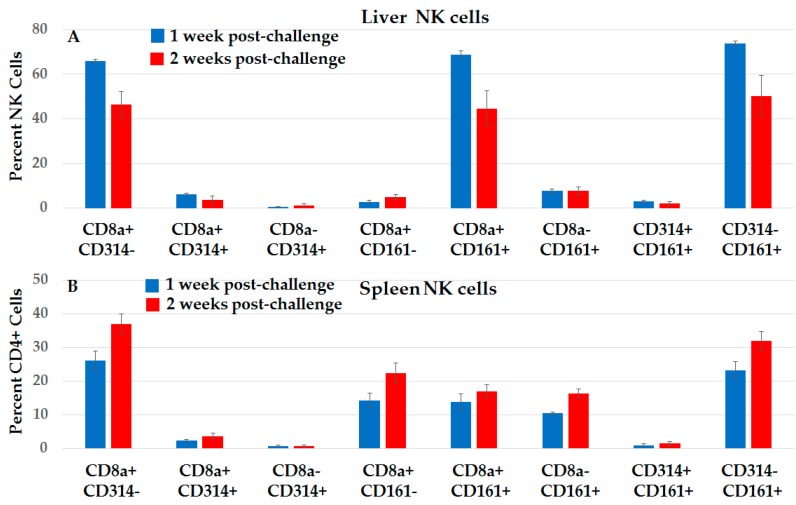
Liver and spleen NK cell subset phenotypic analysis post-challenge shows a vaccine-like effect. Lymphocytes were isolated from liver (**A**) and spleen (**B**) at 1 and 2 weeks post challenge and then stained with CD56, CD3, CD8a, CD314 (NKG2D) and CD161. Analysis done on CD56+/CD3− cells. *n* = 3; error bars = SEM.

Figure 11 shows CD3− CD56+ NK subsets in the liver (11A) and spleen (11B) in the first and the second week after protected rats were challenged. The immune profile in the post-challenge liver looks very different from the NK profile after initial NPS treatments (Figure 6). Unlike post-treatment levels, NPS-induced NK cells that expressed increased CD8+ CD314-NKG2D+ were absent after challenge. The post-challenge liver was now occupied primarily by CD8+ CD161+ NK subsets. These phenotypic characteristics were also present in spleen, except at lower levels. The spleen had CD8+ CD161− and CD8− CD161+ subsets, which were not elevated in the liver. The CD314+ NK subsets that were present after initial treatment were absent after challenge.

### 2.4. Analyses of NPS-Induced Response in Whole Animals

#### 2.4.1. NPS Induces a N1-S1-Specific Long-Lasting Post-HCC Ablation Vaccine-Like Effect in Rats

To determine if the NPS post-ablation vaccine-like effect was specific to N1-S1 HCC, 7 weeks after NPS treatment, tumor-free rats were orthotopically challenged with N1-S1 cells or with rat H4IIE HCC cells. Three weeks later, N1-S1 tumor cells had not formed tumors. In contrast, H4IIE cells induced tumor cell growth not as a defined tumor mass like that observed for N1-S1 tumors, but as tumor cells infiltrating into, but distinctly different from, normal liver parenchyma. This demonstrated that the protective, vaccine-like effect after N1-S1 ablation is specific to N1-S1 HCC.

To determine the longevity of the NPS post-ablation vaccine-like effect in rats with orthotopic N1-S1 HCC [21], a study with 6 rats was carried out over 11 (4 rats) to 15 (2 rats) months from tumor initiation to study end (see materials and methods). Four rats were challenged at 8 weeks and two others at 14 and 19 weeks after treatment. None of these rats had developed tumors. These rats were then boosted twice two months later, and then orthotopically challenged 6–7 months after booster injection. None of these rats established tumors. The vaccine-like effect and granzyme B expression in the NPS treated TME [21] suggested an immune response, as demonstrated in this study. 

#### 2.4.2. NPS Induces a Limited Abscopal Effect

Two different abscopal models were analyzed in the rat N1-S1 model. When one-week-old orthotopic tumors in the medial lobe (148.8 ± 69.3 mm^3^, *n* = 6) were treated and a second orthotopic tumor cell bolus in the left lobe was initiated at the same time (same cell number as primary tumor), three weeks later the treated tumors had regressed (28.4 ± 16.1 mm^3^) and the second tumor was not established (0 mm^3^). This indicates that when a second tumor has escaped the primary site and is seeded as in a micrometastasis at a distant site, NPS treatment of the primary tumor can prevent growth of the distant second tumor.

When a primary tumor in the medial lobe (163.6 ± 23.9 mm^3^) and a secondary tumor in the left lobe (59.6 ± 21.9 mm^3^) were established at the same time (*n* = 6), and the primary tumor was treated one week later, two weeks after treatment, the primary tumor had regressed (107.4 ± 30.5 mm^3^), but the secondary tumor had progressed (3182.5 ± 512.4 mm^3^).

Thus, NPS initiates an immune response from a primary tumor treatment that is sufficient to prevent the growth of a distant tumor while it is initiating, but not once the tumor is established. NPS treatment of primary tumors ablates those tumors, resolves the immunosuppressive TME and initiates an immune response; however, that immune response cannot resolve a tumor that has already established an immunosuppressive TME.

## 3. Discussion

The studies presented here provide a general overview of how the innate and adaptive immune system in rats with orthotopic N1-S1 HCC tumors respond before and after NPS treatment, and how NPS induces a vaccine-like effect that prevents recurrence when orthotopically re-challenged with N1-S1 HCC cells. Immune responses are of interest because NPS treatment itself essentially vaccinates animals in vivo. The success rates of re-challenge are essentially 100%. In a previous study, 21 of 21 rats were protected [21], and scores of rats were protected during this study; protection lasted for as long as 8 months. The NPS-induced vaccine-like effect is not unique to HCC or to rats. It was also observed after NPS ablation of 4T1-Luc mammary cancer tumors in mice [23]. Vaccinations in these rats were specific to N1-S1 HCC tumors; challenge with rat H4IIE HCC induced liver tumors in all 4 rats tested. These studies demonstrate that the protective, vaccine-like effect following NPS ablation [21] is mediated by multiple immune mechanisms appearing after NPS treatment involving elimination of the immunosuppressive TME combined with both adaptive and innate immune mechanisms with influxes of adaptive memory T-cells and specific subsets of NK and NKT-cells expressing activation phenotypes. Although the NPS-induced immune-mediated vaccine-like effect does not specifically constitute typical immunotherapy, NPS does achieve a major immunotherapy goal in exploiting adaptive and importantly innate immune cells in order to destroy tumors [28].

The inept immune response from immune cells with activation markers in the untreated HCC TME was due in part to significantly elevated Tregs (Figure 3A). Although not determined here, Tregs are known to secrete immuno-inhibitory cytokines, such as IL-10, IL-35 and TGFβ, and interact with DCs in the TME that inhibit T-cell effector functions, induce T-cell anergy, prevent T- and B-cell proliferation, and inhibit NK, DC and macrophage anti-tumor functions [29]. The TME plays multiple roles in tumor progression, epithelial-mesenchymal transition and drug resistance, as well as angiogenesis and metastasis [30]. Without NPS treatment, the immunosuppressive TME prevents effective innate and adaptive immune responses, requiring humane euthanasia 2–3 weeks after tumor initiation due to tumor burden [21].

NPS treatment changes the immunosuppressive sequela in the TME and effectively eliminates HCC in these rats by several mechanisms. NPS induces regulated cell death (RCD) in the TME [21], which is known to induce immunogenic cell death (ICD) [31,32,33,34]. To eliminate tumors and induce an immune response, it is surprising that such intense conditions (50 kV/cm, ~10 MW), require 1000 pulses in rat HCC [21, this study] and mouse mammary cancer tumors [23]. However, this includes only 0.1 msec of NPS exposure time. Yet afterwards, Tregs regress and DCs increase (Figure 3) for antigen recognition. In the metastatic 4T1 mammary cancer model, NPS treatment significantly decreases Tregs and myeloid-derived suppressor cells (MDSCs) in the TME and blood [23]. NPS does not selectively eliminate tumor cells in the TME. Tumor cell death is also promoted by death of tumor stroma tissues such as host somatic cells and macrophages, which promote tumor growth, stimulate immune suppression, and interact with cancer stem cells, contributing to tumorigenesis, drug resistance and metastasis [35]. Since NPS eliminates significant cell numbers in the TME, immune cells present after the first few days most likely represent some surviving cells and, more importantly, the influx of cells from the surrounding liver tissue. These are first represented by NK and NKT-cells that expressed CD314-NKG2D and CD161 activation receptors, presumably with cytotoxic activities as shown by expression of granzyme B after treatment [21].

For analyses of NK and NKT-cell phenotypes with CD3 and CD56 or CD161, only CD3+ CD161+ NKT-cells showed NPS-induced elevation (Figure 5). However, a closer phenotypic investigation indicated that NPS specifically induced NK and NKT-cell subsets with CD8+ and CD314-NKG2D and/or CD161 (Figure 6). These cell subsets likely played an important role in HCC tumor elimination after NPS, as well as bridged the gap between innate and adaptive immunity. Given that CD8+ CD314-NKG2D NK and NKT-cells (Figure 6) were only present after treatment, but not after tumor-free rats were challenged (Figure 11), and that NKG2D is the most important NK activation receptor expressed in NK cells regarding virus-infected cells and tumor-cell recognition based on expression of stress ligands on target cells [27], it is likely that NPS induced NK-recognized “stress” ligands during tumor cell death. Some NK cell ligands have been identified in humans and mice [27], but none in rats. Known CD314-NKG2D ligands are all homologous to MHC I class molecules and are either transmembrane proteins or are linked to glycosylphosphatidylinositol [27]. When stress is defined as tumorigenesis, infection or injury, there are several common factors that can activate NKG2D receptors, including DNA damage responses, programmed cell death, upregulation of heat shock pathways, unfolding protein response, oxidative stress, and inflammation. After NPS, tumor cells are severely distressed, but do not die immediately. DNA damage and active caspases can be seen hours after treatment [21,24]. These stress responses also cause the release of ICD molecules [23,24] and molecules with DAMPs that activate pattern recognition receptors (PRRs), such as Toll-, NOD- and RIG-like receptors (TLR, NLR, RLRs). Recognition of hepatitis C virus by TLRs [36] and dsRNA after viral infection by RLRs [37] induces expression of various N KG2D ligands, raising the possibility that signaling through PRRs can induce expression of NKG2D ligands. 

NK cells do not express T-cell or B-cell antigen receptors. Instead, NKs integrate fixed numbers of inhibitory and activating receptors that determine NK cytotoxicity and cytokine release [38]. NK cells can be activated by at least two different mechanisms. To be functional, NKs must first be “licensed” by binding self MHC class 1 molecules with inhibitory receptors. When MHC class I molecules are down-regulated on tumor cells to avoid T-cell recognition, this “missing self” receptor disengages inhibition signaling, slanting the balance toward activating signals [39,40]. Simultaneously, NK receptors identify “stress ligands” or “altered self” molecules that are not present in normal cells, but are upregulated in transformed cells. These are readily recognized and activated in NK cells expressing CD314-NKG2D stimulatory receptors [27]. Engagement of the CD314-NKG2D receptor is known to overrule inhibitory receptors and activate NK cells [27]. In this study, inhibitory receptors were not characterized, primarily because antibodies are not available for these receptors in rats.

Since the liver is an innate immune organ [14], analysis of immune response to HCC in the liver was of specific interest, especially for first-responding NK cells. Rat NK cells are not well-characterized, but mice NKs are in Group 1 of three innate lymphoid cell groups (ILCs) based on cytokine production and transcription factors (TFs) regulating development and functions [41]. The TFs Eomes and T-bet are regulated differently in bone marrow and liver, giving rise to two distinct ILC group 1 NK sub-groups [42]. Fully functional NKs develop in fetal mouse liver and remain in adults, suggesting distinct liver NK precursor cells [43]. Rat NKs have been characterized primarily by morphology. There are “liver-specific” large granular lymphocytes (LGL) called pit cells, which are equivalent to mouse liver NKs [44]. These cells were further characterized as low-density (LD) and high-density (HD) pit cell/NK cells in liver. The LD NKs are akin to activated NKs as early defense against infections and cancer [45,46,47], and are likely related to NPS-activated NKs expressing CD314-NKG2D and CD161 (NK1.1) receptors. Human and rat hepatic NK cells lyse tumor cells that are resistant to splenic or blood NK cells [48], indicating that liver NKs are different from circulating or splenic NKs, but are likely recruited from blood and “educated” in liver to provide distinct functions. While our study did not compare liver to blood or spleen NKs, we did show that tumor-bearing NKs are phenotypically different from naïve NKs (Figure S2), and NPS liver NKs are different from tumor-bearing NKs before NPS treatment (Figure 6). It is likely that transient presence of liver NKs expressing CD8 with CD314-NKG2D and/or CD161 activation receptors are recruited to, “educated” in and activated by NPS in the liver. 

The immune response in vaccinated rats to orthotopic challenge injections presented a somewhat different immune landscape than that seen after NPS treatment. While there were variable increases in CD4+ and CD8+ Tem and Tcm cells in blood, spleen and liver (Figure 7, Figure 8 and Figure 9) after treatment, there were significant increases in CD4+ MPECs and CD4+ and CD8+ SLECs in blood (Figure 7) that were not present after treatment. These MPEC were also elevated in the liver and spleen. This response is similar to the resolution of an acute infection when T-cells differentiate into MPECs expressing CD127 and elevated levels of BCL2 that are destined to provide long-term memory and SLECs expressing KLRG1 that have been repeatedly stimulated by antigens and undergo terminal differentiation and cellular senescence [49,50]. However, the responses here were more apparent in the CD4+ population, while CD8+ MPECs were essentially absent. Nevertheless, after challenge injections, CD8+ lymphocytes were cytotoxic against N1-S1 cells when they were treated with NPS (Figure 10). Also of interest was the absence of NK and NKT-cells expressing CD8 and CD314-NKG2D phenotype in liver after challenge, which was specifically elevated after NPS treatment. In contrast, NK and NKT-cells expressing CD8 and CD161 were specifically elevated after treatment and present after challenge. The CD161 receptor on T-cells can act as a co-receptor defining a specific T-cell subset [51] that secrete IL-17, which among other functions, recruits and activates granulocytes to promote inflammation [52]. The CD161 is also present on memory T-cells, suggesting that CD161+ NK and NKT-cells could uniquely carry memory immune mechanisms [26]. NK cells [53,54] and other innate immune cells [55,56] have recently been shown to be capable of immunological memory.

Other methods using electric fields, including electrochemotherapy (ECT) with bleomycin and irreversible electroporation with calcium recently induced similar vaccine-like effects in murine CT26 colon carcinoma cells [57], but the immune mechanisms remain to be investigated. Furthermore, ECT with bleomycin induced a systemic immune response in a melanoma patient [58]. Here, we characterize the NPS-induced adaptive immune responses in blood, spleen and liver, which lasts for months in rats treated for HCC. Robust innate immune response is induced specifically by NPS in liver NK and NKT-cells expressing dominant activation receptors CD8+ CD314-NKG2D and/or CD161. The immune response after orthotopic challenge is distinct compared to after treatment with enhanced involvement of MPECs and SLECs and CD8+ NK and NKT-cells with an absence of CD314-NKG2D but a presence of CD161. An abscopal response is present in less well-established tumors, indicating that in established tumors, an immunosuppressive TME can become dominant in spite of the presence of NK, NKT and T-lymphocytes. However, NPS treatment can reserve this suppression and establish innate and adaptive immune response as tumors are regressing to establish the NPS-induced protective vaccine-like effect characterized here in rat HCC and in mouse metastatic breast cancer [23].

## 4. Materials and Methods

### 4.1. Animals and Cell Culture 

Male Sprague Dawley rats (6–8 weeks, Envigo, Huntingdon, UK) were cared for and housed in an Association for Assessment and Accreditation of Laboratory Animal Care International (AAALAC, Frederick, MD, USA)-approved facility, and all procedures were carried out with the approval of the Old Dominion University Institutional Animal Care and Use Committee (IACUC). 

Rat N1-S1 HCC cells (American Type Culture Collection, ATCC, Rockville, MD, USA) were cultured as previously described [21].

### 4.2. The HCC Model and Nano-Pulse Stimulation Treatment

For tumor initiation in the orthotopic model, the median liver lobe was exposed through an incision in the abdomen, and 1 × 10^6^ N1-S1 HCC cells in 50 μL saline were injected under the liver capsule. Seven days later, the tumor-bearing lobe was exposed again, and tumors were either sham-treated (electrodes inserted, not pulsed) or treated with NPS delivering 1000 pulses (1–3 pulses/s) with 100 ns durations and electric field strengths of 48–52 kV/cm. Challenge studies were initiated 6–7 weeks after NPS treatment. Animals were euthanized at various times post-treatment and challenged as indicated in the text and Figures.

A long-term challenge study with 6 rats began by tumor initiation (1 × 10^6^ cells) and treatment 1 week later. Rats were then orthotopically challenged (1 × 10^6^) 8 (*n* = 4), 14 and 19 weeks later. All of these tumor-free rats were then challenged by subcutaneous booster injections of N1-S1 cells at 6 weeks, and again at 8 weeks later (1 × 10^6^) (i.e., booster injections separated by 2 weeks). Subcutaneous injected cells did not form tumors in naïve or NPS-treated rats. Then, 6 months (*n* = 2) and 7 months (*n* = 4) after the last boost injection, rats were orthotopically challenged (1 × 10^6^) a second time. Three weeks after this second challenge injection, rats were humanely euthanized. No tumor growths were found on any livers.

### 4.3. Splenocyte Preparation 

Whole spleens were harvested, minced with scissors in RPMI 1640 supplemented with L-glutamine, 25 mM HEPES and 10% FBS and forced through a 40 μm cell strainer (Fisherbrand, Waltham, MA, USA). Cells were spun (350× *g*, 5 min, 4 °C), pellets resuspended, and washed with 1× PBS. Cells were finally suspended in 10 mL of ACK Red Blood Cell (RBC) Lysis Buffer (KD Medical, Columbia, MD, USA), incubated (dark, room temperature) for 7 min and then diluted with 40 mL of 1× PBS. The cells were spun again, and the pellets were suspended (1 × 10^6^/mL) in flow cytometry FACS buffer (5% fetal calf serum in 1× PBS). Cells were then stained for flow cytometry. 

### 4.4. Blood Preparation 

Blood was collected in 5 mM EDTA tubes from anesthetized rats via jugular veins (~500 μL) or end-of-life cardiac puncture (~1 mL). Blood was diluted 1:1 in PBS and layered on 3mL of Ficoll-Paque™ PLUS (GE Healthcare, Little Chalfont, UK) and spun (400× *g*, 30 min, 4 °C). The interphase containing the lymphocytes was collected and washed with FACS buffer. The cells were washed, and pellets were resuspended in FACS buffer (1 × 10^6^/mL). Cells were then stained for flow cytometry. 

### 4.5. Liver Preparation 

After euthanasia, liver lobes or tumors were harvested and placed in 15 mL complete RPMI 1640. Livers/tumors were minced using scissors and a razor blade (without enzymes) until only small chunks remained. The suspensions were filtered through 200 μm filters (PluriSelect, Leipzig, Germany) and then 40 μm cell strainers. Cells were suspended in 50 mL PBS, spun at (350× *g*, 5 min, 4 °C). The pellet was layered on top of 5 mL of Ficoll-Paque™ PLUS, spun (400× *g*, 30 min, 4 °C), and the lymphocyte layer was collected. Cells were washed and resuspended in FACS buffer (1 × 10^6^/mL). Cells were then stained for flow cytometry.

### 4.6. Flow Cytometry and Antibodies 

All flow cytometry and analysis was done on an Aria FACS Diva flow cytometer equipped with 3 lasers, and all analysis was done with the FACS Diva software. The following antibodies were used in specific phenotypic panels: T-Cell Panel: CD4-APC-Cy7 (Biolegend, San Diego, CA, USA), CD8a-V450 (BD Horizon San Jose, CA, USA), CD44-FITC (ThermoFisher Scientific, Waltham, MA, USA), CD62L-eFluor660 (eBiosciences, San Diego, CA, USA), KLRG1-Biotin (BD Pharmingen, San Jose, CA, USA) with Streptavadin-Alexa Fluor 430 (ThermoFisher Scientific) as the secondary, and CD127-PE (R&D Systems, Minneapolis, MN, USA). NK Cell Panel: CD56-Alexa Fluor 700 (Novus Biologicals, Littleton, CO, USA), CD314-PE (ThermoFisher Scientific), CD3-FITC, CD161-APC, CD8a-PerCP all from Biolegend. DC Panel: CD11c-FITC (Bio-Rad, Hercules, CA, USA), CD86-PE (Biolegend), and MHC Class II-APC (Miltenyi Biotec, Teterow GER). Treg Panel: FoxP3-PE, CD4-APC and CD25-FITC all from Biolegend. All antibodies were used at the company’s suggested concentration or titrated for optimal use. 

### 4.7. Cytotoxicity Assay

Spleens were isolated from Sprague Dawley rats at various times post-treatment and post-challenge. Splenocytes were isolated as described above. CD4 and CD8 T-cells were separated with negative selection using CD4 and CD8 T-Cell Isolation kits (StemCell Technologies, Vancouver, BC, Canada). The cross-contamination purity of a typical preparations of CD4+ and CD8+ lymphocytes is shown in Figure S4. N1-S1 cells were labelled with CellTrace Violet (ThermoFisher Scientific, Waltham, MA, USA) according to manufacturer’s instructions. Various ratios of T-cells to labelled N1-S1 cells (5:1, 10:1 and 30:1) were incubated together at 37 °C, 5% CO_2_ for 24 h. The cells were harvested and stained with Annexin-V (APC) (ThermoFisher Scientific, Waltham, MA, USA) in annexin binding buffer (1× PBS, 10 mM Hepes, 150 mM NaCl, 2.5 mM CaCl_2_) and CaspACE FITC-VAD-FMK (Promega, Madison, WI, USA) at room temperature for 20 min. The cells were washed and propidium iodide was added before running on flow cytometry. Cytotoxicity was indicated.

### 4.8. Statistics

Statistical analysis was done by performing a comparison of means by T-test. Significance was determined by using a *p*-value ≤ 0.05. The use of out-bred Sprague Dawley rats and variabilities among them, determination of cell phenotypes over long periods of time and, in some instances, the inclusion of small number of animals, presented conditions that sometimes did not allow values to reach statistical significance. Nevertheless, there were obvious trends that demonstrated changes that were considered meaningful.

## 5. Conclusions

The studies presented here and the studies by Guo and colleagues [23] are the first reports to demonstrate directly that NPS induces immune memory responses in rats and mice, respectively. Furthermore, these immune responses are multifaceted. In addition to showing that Tem and Tcm cells are present in blood and spleens in both studies, the study here further shows that there is a robust innate immune response in the liver and spleens of rats treated with NPS for HCC. NPS up-regulates subsets of NK and NKT-cells that express CD8+ and CD314-NKG2D that are specific to NPS treatment. In contrast, NK and NKT-cells that express CD8 and CD161 are specifically induced after treatment and present after challenge, suggesting they may represent memory cells. MPECs are also prominent after challenge. NPS also resolves the immunosuppressive TME by decreasing Tregs, as well as MDSC in the study by Guo et al., in TME and blood [23]. Thus, NPS induces both innate and adaptive immune responses in rats that eliminate the primary disease and prevent recurrence.

## Supplementary Materials

The following are available online at www.mdpi.com/2072-6694/10/3/69/s1. Figure S1: Flow cytometry NK cell analysis using CD56 and CD3 as the defining markers, Figure S2: Flow cytometry shows NPS activates CD8+ NKs to initiate immune responses in rat HCC, Figure S3: T-Cell analysis post-NPS show differences of various surface markers in response to NPS, Figure S4: Flow cytometry confirms separation purity of CD4+ and CD8+ T-cells.
