# Supplementary material for: Nano-Pulse Stimulation Ablates Orthotopic Rat Hepatocellular Carcinoma and Induces Innate and Adaptive Memory Immune Mechanisms that Prevent Recurrence
Source: Cancers (Basel). 2018 Mar 13;10(3):69. doi: 10.3390/cancers10030069 (PMC5876644; doi:10.3390/cancers10030069)
Supplement: Supplementary file 1 [file cancers-10-00069-s001.docx]

**Supplementary Materials: Nano-Pulse Stimulation Ablates Orthotopic Rat Hepatocellular Carcinoma and Induces Innate and Adaptive Memory Immune Mechanisms that Prevent Recurrence**

**Brittany P. Lassiter, Siqi Guo and Stephen J. Beebe**


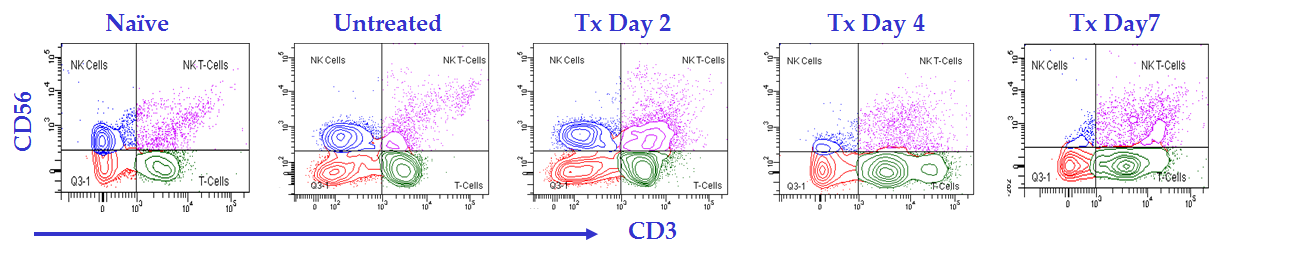


**Figure S1. Flow cytometry NK cell analysis using CD56 and CD3 as the defining markers.** Lymphocytes were isolated from rat liver at various times post-Tx and then stained with the NK cell panel (CD3 and CD56). Results are representative of a typical experiment. These types of analyses formed the basis of Figure 6 with NK cells as CD56+ CD3- (upper left quadrant), NKT-cells as CD56+ CD3+ (upper right quadrant) and T-cells CD3+ CD56- (bottom right quadrant)


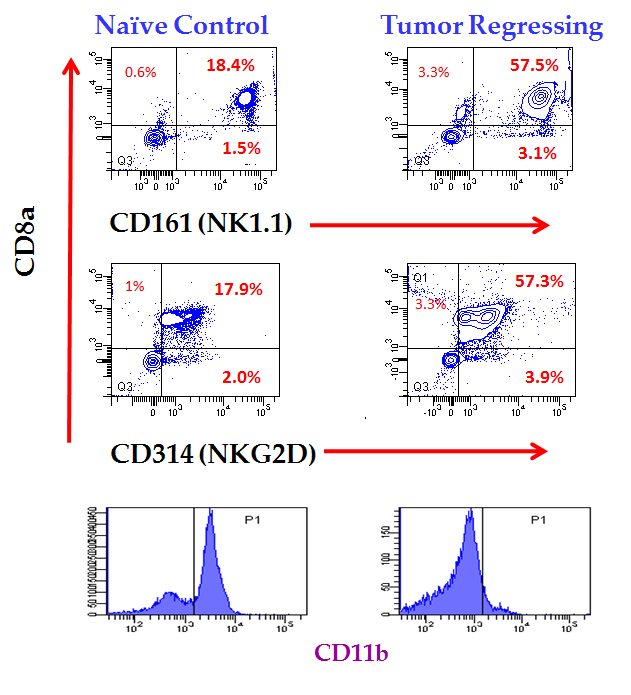


**Figure S2.** Flow cytometry shows NPS activates CD8+ NKs to initiate immune responses in rat HCC. Analysis on CD56+/CD3- NK cells from the liver of a naïve rat and a rats two days after NPS treatment. CD3- CD56+ NK lymphocytes were than analyzed for CD8a, CD161 and CD314-NKG2D as well as CD11b. Results are representative of a typical experiment. This type of analysis formed the basis for data in Figure 7A. An identical analysis was carried out on CD3+ CD56+ NKT-cells for Figure 7B (not shown)


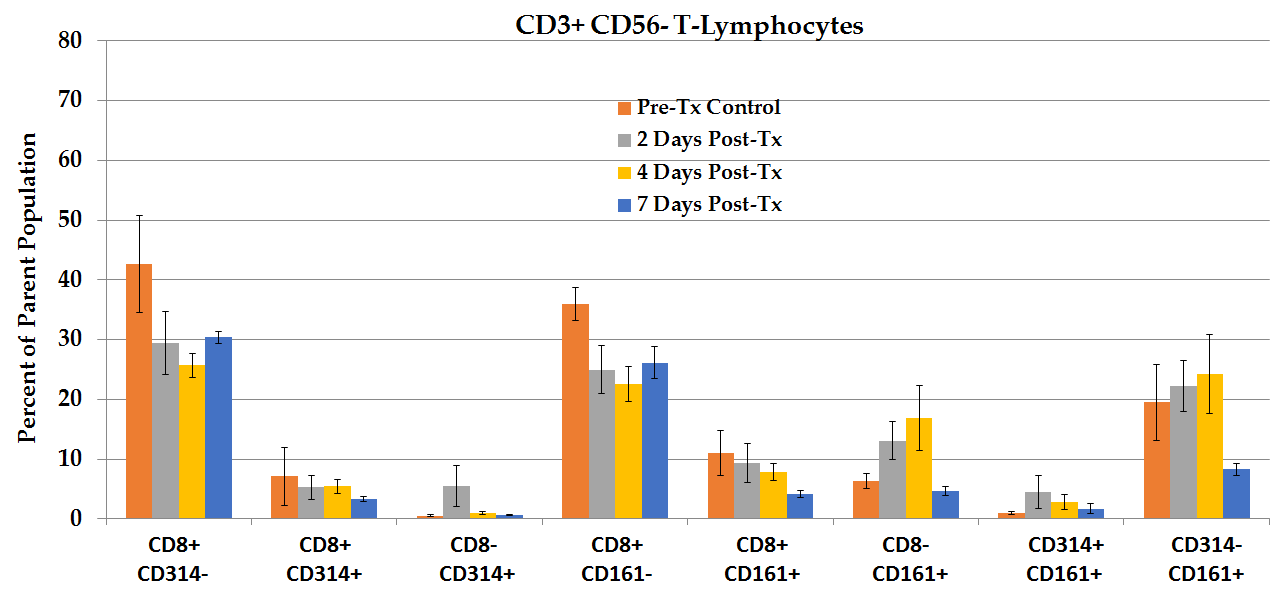


**Figure S3.** T-Cell analysis post-NPS show differences of various surface markers in response to NPS. Liver lymphocyte analysis of CD8a, CD314 (NKG2D) and CD161 on T-cells (CD56-/CD3+) Naïve n=4, Pre-Tx n=5; others n=6; error bars = SEM. These experiments on CD3+ CD56- T-cells were analogous to experiments in Figure 7A on NK cells and Figure 7B on NKT-cells.


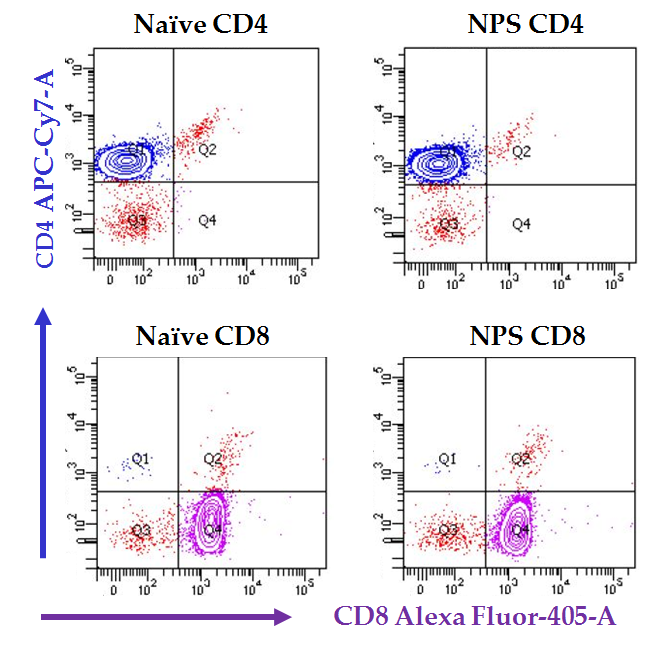


**Figure S4.** Flow cytometry confirms separation purity of CD4+ and CD8+ T-cells. Results confirm purity of T-cell separation kit. The image represents a typical experiment. This CD4+ / CD8+ lymphocyte separation formed the bases for experiments in Figure 3.
